# A Call to Action to Enhance Filovirus Disease Outbreak Preparedness and Response

**DOI:** 10.3390/v6103699

**Published:** 2014-09-30

**Authors:** Paul Roddy

**Affiliations:** Independent Epidemiology Consultant, Barcelona, 08010, Spain; E-Mail: roddypd@gmail.com

**Keywords:** Ebola, ebolavirus, Marburg virus, marburgvirus, *Filoviridae*, filovirus, outbreak, preparedness, response, data collection, treatment, guidelines, surveillance

## Abstract

The frequency and magnitude of recognized and declared filovirus-disease outbreaks have increased in recent years, while pathogenic filoviruses are potentially ubiquitous throughout sub-Saharan Africa. Meanwhile, the efficiency and effectiveness of filovirus-disease outbreak preparedness and response efforts are currently limited by inherent challenges and persistent shortcomings. This paper delineates some of these challenges and shortcomings and provides a proposal for enhancing future filovirus-disease outbreak preparedness and response. The proposal serves as a call for prompt action by the organizations that comprise filovirus-disease outbreak response teams, namely, Ministries of Health of outbreak-prone countries, the World Health Organization, Médecins Sans Frontières, the Centers for Disease Control and Prevention—Atlanta, and others.

## 1. Introduction

Ebola virus disease (EVD) and Marburg virus disease (MVD) in human and non-human primates (NHPs) are caused by seven distinct viruses that produce filamentous, enveloped particles with negative-sense, single-stranded ribonucleic acid genomes. These viruses belong to the *Filoviridae* family and its *Ebolavirus* and *Marburgvirus* genera, respectively [1]. An eighth filovirus, Lloviu virus (LLOV), assigned to the third filovirus genus, *Cuevavirus* has thus far not been associated with human disease [2,3]. To date, no human or NHP infections of LLOV have been known to occur [4]. 

### 1.1. Outbreak Response Teams, Objectives, and Components

For a human filovirus-disease outbreak to be declared, a single laboratory-confirmed case must be identified. If filovirus infection is suspected but not laboratory-confirmed, the possible outbreak remains speculative and is not recognized and declared by the World Health Organization (WHO), the acknowledged authority for international filovirus reporting. As described elsewhere [5,6,7,8], filovirus-disease outbreak response teams (ORTs) typically comprise first-line medical professionals reinforced by the relevant Ministry of Health, the WHO, Médecins Sans Frontières (MSF), the Centers for Disease Control and Prevention—Atlanta (CDC), and others. 

Overall filovirus-disease outbreak response objectives are to (1) prevent and control the spread of the disease and (2) provide infected patients with optimal monitoring and medical treatment. To achieve these objectives, the following outbreak-response components are realized. Within an affected community: (1) epidemiological surveillance for case detection, (2) burial and disinfection, (3) home-based risk reduction, (4) peripheral health-facility support, (5) psychosocial support (6) information and education campaigns, and (7) ecological studies. Within a filovirus ward and health facility: (1) design and construction of the filovirus ward, (2) case diagnosis, (3) case detection in the health facility, (4) case management, (5) psychological care, and (6) infection control in the health facility. 

### 1.2. Response-Component Protocol Modifications

Due to numerous past impediments to efficiency and effectiveness, protocols corresponding to each filovirus-disease outbreak-response component have purportedly been modified for improvement [5,6,9,10,11,12,13,14,15,16,17,18]. These modifications aimed to promote cultural sensitivity, community collaboration, transparency of activities, improved data collection initiatives, and the active involvement of all stakeholders during all phases of the response [7]. 

Further, it is now understood that the acceptability of a filovirus ward in a host community requires that psychological and cultural factors be considered during all stages of filovirus ward planning and implementation, including the provision of optimal medical care, which increases the acceptability of response components within the affected community and may improve survival rates for some patients [15,17,18]. Additionally, as filovirus clinicians often triage patients based on presenting signs and symptoms and contact history, ORTs should now be cognizant of the crucial importance of collecting and analysing high-quality epidemiological and clinical data, which contribute to case definition refinement, and thereby facilitate outbreak control and treatment strategies [5,6,17,18,19].

## 2. Delineation of the Problem

Despite the purported protocol modifications, limitations to efficient and effective filovirus-disease outbreak preparedness and response remain [7,18,20,21]. Thus, ensuing the acknowledgement of challenges inherent to and identification of shortcomings in current outbreak preparedness and response, a proposal for future enhancement is herein provided. A brief overview of human filovirus-disease outbreak frequency, magnitude, and geographic distribution evinces the pertinence of the proposal, while the proposal itself serves as a call for prompt action by Ministries of Health of outbreak-prone countries, the WHO, MSF, CDC, and others. 

### 2.1. Outbreak Frequency and Magnitude

**Figure 1:**
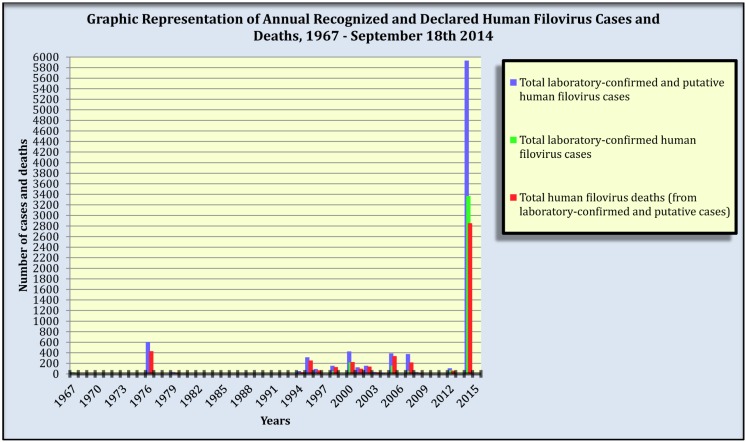
Annual recognized and declared filovirus-disease cases and deaths in humans, 1967–18 September 2014. Note: Biosafety level-4 laboratory accidental filovirus exposures are included as they involve human cases. Filovirus-disease cases and deaths from outbreaks that occurred over multiple years are assigned to their first year of occurrence. Data source: Table 1.

Since the initial 1967 filovirus discovery [22,23], a total of 41 human filovirus-disease outbreaks have been recognized and declared; 29 of these were EVD and 12, MVD; each outbreak occurred in or was thought to have originated from widely distributed areas of sub-Saharan Africa. As of 18 September 2014, these outbreaks have resulted in 8883 laboratory-confirmed or putative filovirus-disease cases and 4921 deaths, yielding a mean case fatality ratio (CFR) of 55.4% [24,25,26,27,28,29,30,31,32,33,34,35,36] (Table 1, Figure 1).

An increase in frequency and magnitude of recognized and declared human filovirus-disease outbreaks have occurred in the recent 1994 to 2014 time period (Table 1, Figure 1). The only two recognized major MVD outbreaks to occur in their natural setting (sub-Saharan Africa) transpired within this period: Durba and Watsa, DRC (1998–2000) and Uige, Angola (2005) [37]. Remarkably, the current outbreaks of 2014 have thus far yielded nearly sixty-seven percent of all recognized and declared filovirus infections known to have occurred since 1967 (Table 1). 

Filovirus-disease outbreaks are currently unpredictable in their timing and, within sub-Saharan Africa, their location [37,38]. The extent to which the recent increase in outbreak frequency can be attributed to improved surveillance and/or laboratory diagnostic capacity rather than an actual increase in number of outbreaks is uncertain. Seroprevalence studies [39,40,41,42,43,44,45,46] suggest that symptomatic and asymptomatic endemic filovirus infections occur, but transmission is typically recognized only when amplified [20,25,47,48]. There is also a suggested high likelihood of unrecognised outbreaks or isolated cases in unmonitored areas [44,46,49,50]. Further research regarding filovirus-disease outbreak frequency and magnitude is warranted. 

### 2.2. Outbreak Geographic Distribution

Although recent research has implicated fruit bats of multiple species as natural reservoirs [46,51,52,53,54,55,56,57], detailed ecology of ebolaviruses and marburgviruses and their complete maintenance cycle are, to date, uncertain and are the subject of ongoing study [37,58,59,60,61]. Nonetheless, high seroprevalence of Ebola virus-specific immunoglobulin G (IgG) in chimpanzees residing in Republic of the Congo, Gabon, and Cameroon [44], and bats from Republic of the Congo and Gabon [46] suggest that Ebola virus circulates continuously and with long-term persistence in tropical forest regions of sub-Saharan Africa, causing lethal and non-lethal infections in human and NHPs [44]. A serological survey in Gabon found an Ebola virus-specific IgG seroprevalence of 15.3% among rural human populations; the highest reported to date, suggesting a common source of human exposure, such as fruit contaminated by bat saliva [58,59]. Furthermore, excluding accidental exposures in biosafety level-4 laboratories, all recognized human filovirus-disease outbreaks to date can be traced back to tropical forest regions and other widely distributed areas of sub-Saharan Africa [20,21,24,36,37]. As of 18 September 2014, human filovirus-disease outbreaks have been laboratory-confirmed and declared in the following sub-Saharan African countries: Republic of the Congo, Gabon, Zaire (and present-day Democratic Republic of the Congo), Rhodesia (present-day Zimbabwe), South Africa, Kenya, Angola, Côte d’Ivoire, Uganda, Sudan (present-day South Sudan), Guinea, Liberia, Sierra Leone, Nigeria, and Senegal (Table 1) [24,36].

**Table 1:** Recognised and declared filovirus-disease outbreaks in humans (1967–18 September 2014). Note: Biosafety level-4 laboratory accidental exposures are categorized as filovirus-disease outbreaks as they involve human cases.

Number	Year	Filovirus	Outbreak Location	Laboratory Confirmed Cases	Putative Cases	Total cases (Laboratory Confirmed Plus Putative)	Deaths	CFR* (%)
1	1967	Marburg virus	Marburg and Frankfurt, West Germany and Belgrade, Yugoslavia	23	9	31	7	22.6
2	1975	Marburg virus	Johannesburg, South Africa (Imported from Rhodesia)	3	0	3	1	33.3
3	1976	Sudan virus	Maridi and Nzara, Sudan	§	§	284	151	53.2
4	1976	Ebola virus	Yambuku, Zaire	§	§	318	280	88.1
5	1976	Sudan virus	Porton, United Kingdom laboratory accident at the Microbiological Research Establishment	1	0	1	0	0.0
6	1977	Ebola virus	Tandala, Zaire	1	0	1	1	100.0
7	1979	Sudan virus	Nzara, Sudan	2	32	34	22	64.7
8	1980	Marburg virus	Kisumu and Nairobi, Kenya	2	0	2	1	50.0
9	1987	Ravn virus	Mombasa, Kenya	1	0	1	1	100.0
10	1988	Marburg virus	USSR laboratory accident	1	0	1	1	100.0
11	1990	Marburg virus	USSR laboratory accident	1	0	1	0	0.0
12	1994	Ebola virus	Mékouka, Ogooué-Ivindo Province, Gabon	7	45	52	32	61.5
13	1994	Taï Forest virus	Taï Forest, Côte d’Ivoire (Treated in Switzerland)	1	0	1	0	0.0
14	1995	Ebola virus	Kikwit, Zaire	§	§	315	254	80.6
15	1996	Ebola virus	Mayibout, Ogooué-Ivindo Province, Gabon	3	28	31	21	67.7
16	1996–1997	Ebola virus	Booué, Ogooué-Ivindo Province, Gabon	6	54	60	45	75.0
17	1996	Ebola virus	Johannesburg, South Africa (Imported from Gabon) [linked to 1996-97 Booue, Gabon outbreak]	1	1	2	1	50.0
18	1998–2000	Marburg virus. Ravn virus	Durba and Watsa, Democratic Republic of the Congo	51	103	154	128	83.1
19	2000–2001	Sudan virus	Gulu, Uganda	218	207	425	224	52.7
20	2001–2002	Ebola virus	Ogooué-Ivindo Province, Gabon and Cuvette Ouest Region, Republic of the Congo	§	§	124	97	78.2
21	2002	Ebola virus	Ogooué-Ivindo Province, Gabon and Cuvette Ouest Region, Republic of the Congo	§	§	11	10	90.9
22	2002–2003	Ebola virus	Kellé, Cuvette Ouest Region, Republic of the Congo	2	141	143	128	89.5
23	2003	Ebola virus	Mbandza Mbomo, Cuvette Ouest Region, RC	§	§	35	29	82.9
24	2004	Ebola virus	Koltsovo, Russian Federation laboratory accident at the State Research Center of Virology and Biotechnology (Vector)	1	0	1	1	100.0
25	2004	Sudan virus	Yambio, Sudan (currently South Sudan)	§	§	17	7	41.2
26	2005	Ebola virus	Etoumbi, Republic of the Congo	1	11	12	9	75.0
27	2004–2005	Marburg virus	Uíge, Angola	158	216	374	329	88.0
28	2007	Ebola virus	Kasai Occidental Province, Democratic Republic of the Congo	21	202	223	179	80.3
29	2007	Marburg virus , Ravn virus	Kamwenge, Uganda	4	0	4	1	25.0
30	2007–2008	Bundibugyo virus	Kikyo and Bundibugyo, Uganda	30	119	149	37	24.8
31	2008	Marburg virus	Holland (Imported from Uganda)	1	0	1	1	100.0
32	2008–2009	Ebola virus	Mweka and Luebo health zones in the Province of Kasai Occidental Province, Democratic Republic of the Congo	§	§	32	15	46.9
33	2008–2009	Marburg virus	USA (Imported from Uganda)	1	0	1	0	0.0
35	2011	Sudan virus	Luwero District, Uganda	1	0	1	1	100.0
36	2012	Marburg virus	Kabale, Ibanda, Mbarara, and Kampala, Uganda	15	8	23	9	39.1
37	2012	Sudan virus	Kagadi, Kibaale District, Uganda	11	13	24	17	70.8
38	2012	Bundibugyo virus	Isiro and Viadana, Haut-Uélé District, Province Orientale, Democratic Republic of the Congo	36	16	52	25	48.1
39	2012	Sudan virus	Luweero District, Uganda	6	1	7	4	57.1
40**	2014 (as of 18 Sept. 2014)	Ebola virus	West Africa (To date: Guinea, Liberia, Sierra Leone, Nigeria, and Senegal) Cases exported to USA, Spain, and others were recorded in the country where the transmission occurred.	3341	2523	5864	2811	47.9
41**	2014 (as of 18 Sept. 2014)	Ebola virus	Boende District, Equateur Province, Democratic Republic of the Congo	28	40	68	41	60.3
**Total**				3979	3769	8883	4921	55.4

* CFR = case fatality ratio = deaths divided by total cases. ^§^ Although an undetermined number of cases were stated to be laboratory-confirmed by the World Health Organization and/or peer-reviewed and published scientific articles, the precise number of confirmed *versus* putative cases was not mentioned. ** Outbreak is ongoing at time of publication of this paper.

### 2.3. Challenges and Shortcomings in Outbreak Preparedness and Response 

#### 2.3.1. Challenges 

The 2014 EVD outbreak in West Africa (To date: Guinea, Liberia, Sierra Leone, Nigeria, and Senegal) [20,36,62,63,64,65] prompts recollection of some of the inherent, formidable, and reoccurring challenges filovirus ORTs experience when implementing disease control and treatment strategies in geographically dispersed communities served by antiquated health systems [62,66]. For example, to effectively manage and implement response components, teams must continuously replenish their numerous multidisciplinary and multisectoral human resources, who routinely operate in remote locations [17,36,62,67,68,69]. Response efforts are further complicated when components are suspended or diminished ensuing community resistance to the intervention due to fear of the disease and misconception of outbreak response objectives and components, as transpired in Gabon in 2002 [11], the Republic of the Congo in 2002 and 2003 [11,12,13], Angola in 2005 [6], and West Africa in 2014 [66,70,71,72,73]. Finally, as seen in previous outbreaks [74] and most recently in 2014 in Guinea [20] and the Democratic Republic of the Congo [75], another challenge to filovirus-disease outbreak response includes the weeks or months of habitually unrecognized secondary transmission occurring in a community prior to the recognition and declaration of the outbreak, which contribute to high filovirus-disease case numbers and wide geographic spread [20,36]. 

ORTs diligently work to overcome these and other challenges, in part by sensitising affected communities about filovirus disease, transmission routes, and outbreak response objectives and components. Notwithstanding, below is a non-exhaustive list of identified and current filovirus-disease outbreak preparedness and response shortcomings, followed by a proposal aimed at Ministries of Health of outbreak-prone countries, the WHO, MSF, CDC, and others to consider for enhancing future efforts. 

#### 2.3.2. Shortcoming #1—Data Collection Initiatives

##### 2.3.2.1. Epidemiological Data

Filovirus-disease outbreaks continue to be plagued by poor epidemiological and clinical data collection initiatives. Surveillance teams typically use epidemiological data to identify and follow-up primary and/or secondary transmission contact links [37,76,77,78,79], an essential outbreak control activity [9]. Contact tracing databases such as the WHO Field Information Management System (FIMS) [80], schematic secondary transmission-chain representations of epidemiological contact-tracing investigations [20], and/or an Epi Info™ application recently designed by the CDC [81,82] have been created to facilitate these efforts.

Regrettably, these databases currently lack involved inter-organizational ownership, regularly scheduled user training, and—particularly when data comprise patient demographic, epidemiological, and clinical variables—data-sharing agreements approved by the ethical review mechanisms of each ORT organization, including the relevant Ministries of Health. These lacunae have likely contributed to the intermittent employment of FIMS in filovirus-disease outbreak settings since its 2005 inception and to the inter-outbreak methodological variance in schematic secondary transmission-chain representations. Also, despite its stated potential for inter-agency communication and data management efficiency, as well as its epidemiology, laboratory, clinical, and mapping module design input received from individual members of the WHO, MSF, and the Uganda Ministry of Health, and subsequent pilot testing [82], the effectiveness of the proposed Epi Info™ application would likely be impeded by the non-proficiency in Epi Info™ among an ORT’s high human-resource numbers and turn-over rate [62,68,69]. Onsite Epi Info™ training conducted near the end of an outbreak, when incidence rates have abated, would largely be ineffective for facilitating control efforts for that particular outbreak, while training sessions conducted during the height of an outbreak would be quixotic and inadvisable [6,18] as ORT members are responsible for and immersed in a multitude of intervention activities, leaving insufficient time to attend software training sessions. Ideally, relevant inter-organizational ORT members from relevant Ministries of Health, the WHO, MSF, CDC, others would receive regularly scheduled database training between outbreak occurrences and deploy to outbreak settings with the required software proficiency.

Despite the current lacunae, these databases facilitate outbreak control, and their future use is encouraged. However, outbreak control efficiency and effectiveness can be strengthened through inter-organizational preparedness, which would remove a multidisciplinary and multisectoral ORT’s dependence on a single organization to manage and analyze epidemiological and clinical data for real-time, intra-outbreak decision making. Ministries of Health of outbreak-prone countries and international ORT organizations must foster involved ownership, commit to regularly scheduled human-resource training, particularly between outbreak occurrences, and ensure the ethical use of patient data. 

##### 2.3.2.2. Clinical Data

Filovirus-disease clinical data-collection initiatives in human outbreak settings have consistently yielded low-quality data and few peer-reviewed published analyses to contribute knowledge of these poorly understood diseases. Moreover, to date, despite the same organizations responding to all 24 recognized human filovirus-disease outbreaks that have occurred in sub-Saharan Africa since 1995 (Table 1), clinical data have not been systematically collected; habitually fail to record patients’ symptom onset, frequency, and duration; are often obtained without written and informed patient or caregiver consent [18,20]; and lamentably, for many outbreaks, not collected at all. Stated previously [15,17,18,83], and with continued relevance today, concise yet thorough data collection guidelines, templates, training, and armamentarium, similar to those used for intensive care patients in industrialized countries, must be prioritized through inter-organizational preparedness initiatives prior to the next outbreak occurrence and beyond. 

#### 2.3.3. Shortcoming #2—Evidence-based Case Management 

Coupled with the feasibility of provision in an outbreak setting and an affected community’s values and preferences, optimal filovirus-disease medical care should be defined by methodologically sound, patient-centered clinical research [84,85,86,87]. However, to date, best practice for filovirus-disease case management is primarily based on anecdotal evidence, while the impact of supportive and/or innovative treatment on clinical outcome is unknown [17]. Moreover, few scientific studies have been designed and implemented to critically evaluate treatment effectiveness. Beyond the current primary focus on filovirus-disease containment [21], ORTs must aim to apply an appropriate and Ethical Review Board-approved study design for the collection and analyses of high-quality epidemiological and clinical data to generate improved evidence for supportive and/or innovative treatment effectiveness in human outbreak settings [17].

#### 2.3.4. Shortcoming #3—Outbreak Preparedness and Response Guidelines

In 1998, together with contributions from over 20 relevant organizations, the CDC and the WHO jointly produced an infection control manual for viral haemorrhagic fevers in the African health care setting [9]. Later, in 2008, the WHO produced an interim summary of infection control recommendations when providing care to filovirus patients [88]. That same year, MSF developed an internal filovirus-disease outbreak-response guideline to provide relevant MSF staff with a practical summary of filovirus-disease intervention objectives, activities, and lessons learned from previous outbreaks [89]. The MSF internal guideline summarized a draft of what was meant to be the official MSF Filovirus Outbreak Control Guidance Manual; to date this manual has not yet been completed. Most recently, during the 2014 EVD outbreak in West Africa, the WHO released an interim guideline for filovirus-disease outbreak preparedness, alert, control, and evaluation [90], an interim infection-prevention and control guideline for the care of patients with filovirus disease [91], and a guideline for the clinical management of patients with viral haemorrhagic fever [92]. 

Collectively, the above-mentioned filovirus guidelines are informative and advise ORT control and treatment strategies. Notwithstanding, the technical content provided in these guidelines, particularly with respect to filovirus epidemiology, ecology, data collection templates and procedures, information and education campaigns, case definitions, laboratory diagnoses, treatment, and lessons learned, all require further elaboration, improvement, harmonization, and updating, ideally prior to the next outbreak occurrence. Ministries of Health of outbreak-prone countries, the WHO, MSF, CDC, and others would greatly enhance the efficiency and effectiveness of their filovirus-disease outbreak preparedness and response if they collaboratively developed and implemented a technically sound, comprehensive, and updated inter-organizational guideline that incorporates scientific and technical advances since 2008 and responds to the increasing expectation on their ORTs to improve their data collection and case management strategies [18,21,69]. 

#### 2.3.5. Shortcoming #4—Surveillance in Outbreak-prone Countries

In sub-Saharan Africa, antiquated health systems and the non-ubiquity of filovirus-disease surveillance mechanisms, trained human resources, and diagnostic capacity all contribute to the paucity of functioning filovirus-disease surveillance systems. The 2014 EVD outbreak in West Africa has demonstrated, once again, that filovirus-disease outbreaks are often unpredictable in their timing and, within sub-Saharan Africa, their location [37,38]; unrecognized—particularly in unmonitored areas [44,46,49,50]; and undiagnosed until disease amplification occurs in a health facility and/or community, often weeks or months after secondary transmission commenced [20,25,39,40,41,42,43,44,45,46,47,48,75,93,94]. 

Yet, outbreak control and treatment efforts are facilitated when an intervention follows early recognition of disease transmission [7,9]. ORTs must initiate discussion on how to best create and sustain a functional local, national, regional, and/or international filovirus-disease surveillance network in outbreak-prone countries, a formidable yet plausible endeavor. Established high-containment laboratories in filovirus-disease outbreak-prone countries that strengthen local and national surveillance and perform filovirus and differential diagnostics, pathogenesis, ecological research, and more, such as the International Center for Medical Research in Franceville, Gabon (CIRMF) [95], the Uganda Virus Research Institute (UVRI) and CDC in Entebbe, Uganda [96], mobile laboratory units [97,98], and others may provide guidance for creating, implementing, and sustaining functional filovirus-disease surveillance systems. Additionally, relevant Ministries of Health have recently adopted regulations [99], strategies [100], and frameworks [7,101], and created national and regional surveillance initiatives [102,103], which should inform these efforts. 

## 3. Proposal for Enhancing Filovirus-Disease Outbreak Preparedness and Response 

To date, within ORTs no single individual or group has been able to devote the time and resources required to adequately advance filovirus-disease data-collection initiatives, evidence-based case management, comprehensive outbreak-response guidelines, international surveillance networks, and more. Between outbreaks, when thorough preparedness and research endeavours are ideally conducted, ORT members are occupied with other non-filovirus-disease-related components of their employment. As such, much of the preparedness and research for the benefit of the efficiency and effectiveness of future outbreak response is forsaken. 

It is proposed that, in accordance with the principles of the ethical practice of public health [104] and research [105], and previously suggested frameworks [17,25], the Ministries of Health of outbreak-prone countries, the WHO, MSF, CDC, and others promptly create and sustain a functional, multisectoral, and multidisciplinary filovirus-disease working group to advance outbreak preparedness and response efforts. The pertinence of such a working group is underscored by the recent increase in frequency and magnitude of recognized and declared human-filovirus disease outbreaks and the potential ubiquity of pathogenic filoviruses throughout sub-Saharan Africa. Critical components of this proposal include the following.

1. As the WHO is responsible for providing leadership on global health matters, shaping health research agendas, setting norms and standards, articulating evidence-based policy options, providing technical support to countries, and monitoring and assessing health trends [106,107] it should convene, coordinate, and ensure maintained funding for a functional filovirus-disease working group.
2. The filovirus-disease working group would promptly convene to define its objectives, activities, timelines, and expected outputs and deliverables.
3. The proposed overall objective of the filovirus-disease working group would be to develop and implement frameworks, guidelines, protocols, templates, and trainings for efficient and effective filovirus-disease outbreak preparedness and response.
4. The participating members of the filovirus-disease working group would collaboratively address the preparedness and response shortcomings identified in this paper and elsewhere.
5. All patient data collection and analysis initiatives would prioritize and seek the guidance and approval of relevant Ethical Review Boards.
6. Core members of the filovirus-disease working group should be agile and resilient to be deployed immediately on outbreak recognition [17].
7. Proposed outputs and deliverables of the filovirus-disease working group include, but are not limited to, the development and implementation in outbreak settings of the following.
  - Protocols, templates, and trainings that ensure the collection and analyses of high-quality epidemiological and clinical data;
  - Study protocol(s) with an appropriate evaluation scheme for evidence-based case management;
  - Inter-organizational, technically-sound, comprehensive, and updated, outbreak-response guidelines;
  - Framework(s) for a filovirus-disease surveillance network in outbreak-prone countries, including corresponding guidelines, inter-organizational agreements, protocols, trainings, and templates.

## 4. Conclusion

With uncertain timing and location in sub-Saharan Africa, frequent and high magnitude filovirus-disease outbreaks will likely occur again. The idea of improving filovirus-disease outbreak preparedness and response must therefore be embraced. This paper stands as a call for prompt action by Ministries of Health of outbreak-prone countries, the WHO, MSF, CDC, and others to create and sustain a functional, multisectoral, and multidisciplinary filovirus-disease working group to address the inherent challenges and current shortcomings in filovirus-disease outbreak preparedness and response, which would greatly enhance future efforts. 
